# Supplementary material for: The Eukaryotic Translation Initiation Factor 4F Complex Restricts Rotavirus Infection via Regulating the Expression of IRF1 and IRF7
Source: Int J Mol Sci. 2019 Mar 29;20(7):1580. doi: 10.3390/ijms20071580 (PMC6480131; doi:10.3390/ijms20071580)
Supplement: Supplementary file 1 [file ijms-20-01580-s001.pdf]

Article

The Eukaryotic Translation Initiation Factor 4F Complex Restricts Rotavirus Infection via Regulating the Expression of IRF1 and IRF7

Sunrui Chen [1,2,†], Cui Feng [3,†], Yan Fang [4,†], Xinying Zhou [5], Lei Xu [6], Wenshi Wang [2], Xiangdong Kong [3], Maikel P. Peppelenbosch [2], Qiuwei Pan [1,2] and Yuebang Yin [2,*]

1 Biomedical Research Center, Northwest Minzu University, Lanzhou 730030, China; s.chen.1@erasmusmc.nl (S.C.); q.pan@erasmusmc.nl (Q.P.)
2 Erasmus MC-University Medical Center, 3000 CA Rotterdam, the Netherlands; w.wang.2@erasmusmc.nl (W.W.); m.peppelenbosch@erasmusmc.nl (M.P.P.)
3 Department of Materials Engineering, Zhejiang Sci-Tech University, Hangzhou 310018, China; fengc93103@126.com (C.F.); kxd01@126.com (X.K.)
4 College of Basic Medicine, Shannxi University of Chinese Medicine, Xianyang 712046, China; fangyan9494@163.com
5 Institute of Molecular Immunology, School of Laboratory Medicine and Biotechnology, Southern Medical University, Guangzhou 510515, China; xylona14@hotmail.com
6 College of Life Sciences, Northwest A&F University, Yangling 712100, China; xulei@nwafu.edu.cn
† These authors contributed equally to this work.
* Correspondence: tonyerasmusyin@163.com; y.yin@erasmusmc.nl

Supplementary tables:

Table S1. qRT-PCR primers (human gene) used in the study, from 5' to 3'.

Gene name	Sense primer	Anti-sense primer
eIF4A	AAGCCGTGGATTCAAGGACCAG	CACCTCAAGCACATCAGAAGGC
eIF4E	ATGCCTGGCTGTGACTACTCAC	GAGGTCACTTCGTCTCTGCTGT
eIF4G	GCCATTTCAGAGCCCAACTTCTC	CGGAAGTTCACAGTCACTGTTGG
PDCD4	ACTGTGCCAACCAGTCCAAAGG	CCTCCACATCATACACCTGTCC
GAPDH	TGTCCCCACCCCCAATGTATC	CTCCGATGCCTGCTTCACTACCTT

Table S2. shRNA target sequences.

ShRNA	Sequence
sheIF4A-1	CCGGGCCGTGTGTTTGATATGCTTACTCGAGTAAGCATATCAAACACACGGCTTTTTG
sheIF4A-2	CCGGGCCGTAAAGGTGTGGCTATTACTCGAGTAATAGCCACACCTTTACGGCTTTTTG
sheIF4A-3	CCGGCCTTGTATCAAGGGTTATGATCTCGAGATCATAACCCTTGATACAAGGTTTTTG

sheIF4A-4	CCGGCGAAATGTTAAGCCGTGGATTCTCGAGAATCCACGGCTTAACATTTCGTTTTTG
sheIF4E-1	CCGGCCACTCTGTAATAGTTCAGTACTCGAGTACTGAACTATTACAGAGTGGTTTTTG
sheIF4E-2	CCGGCCAAAGATAGTGATTGGTTATCTCGAGATAACCAATCACTATCTTTGGTTTTTG
sheIF4E-3	CCGGCCGACTACAGAAGAGGAGAAACTCGAGTTTCTCCTCTTCTGTAGTCGGTTTTTG
sheIF4E-4	CCGGCGGCTGATCTCCAAGTTTGATCTCGAGATCAAACTTGGAGATCAGCCGTTTTTG
sheIF4G-1	CCGGCCCTACAGAATTTGGGACCTACTCGAGTAGGTCCCAAATTCTGTAGGGTTTTTG
sheIF4G-2	CCGGGCCCTTGTAGTGACCTTAGAACTCGAGTTCTAAGGTCACTACAAGGGCTTTTTG
sheIF4G-3	CCGGGCAGATAGTATCCAACACGTTCTCGAGAACGTGTTGGATACTATCTGCTTTTTG
sheIF4G-4	CCGGCCCAAGTAATGATGATCCCTTCTCGAGAAGGGATCATCATTACTTGGGTTTTTG
shPDCD4-1	CCGGGCGGTTTGTAGAAGAATGTTTCTCGAGAAACATTCTTCTACAAACCGCTTTTTG
shPDCD4-2	CCGGCTGACCTTTGTGGGACAGTAACTCGAGTTACTGTCCCACAAAGGTCAGTTTTTG
shPDCD4-3	CCGGCTACCATTACTGTAGACCAAACTCGAGTTTGGTCTACAGTAATGGTAGTTTTTG

Table S3. Sg RNA Primers (human gene) used in the study, from 5' to 3'.

Sg RNA	Sense primer	Antisense primer
eIF4A	CACCGCCCCCGATACAGGCGTGAC	CGGGGGCTATGTCCGCACTGCAAA
eIF4E	CACCGGGACGTCCCCACTTGTCCG	CCCTGCAGGGGTGAACAGGCCAAA
eIF4G	CACCGCTATCCAGTCGAACACCCGC	CGATAGGTCAGCTTGTGGGCGCAAA

Supplementary Figures:

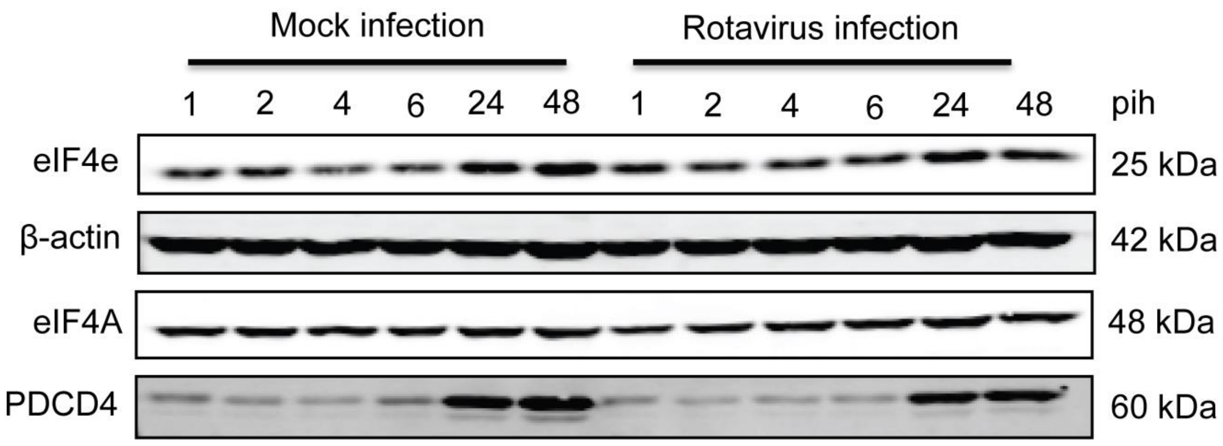

Figure S1. The effect of rotavirus and mock infection (1, 2, 4, 6, 24 and 48 hr) on the expression level of eIF4E, eIF4A and PDCD4. Western blot assay detected the expression of eIF4E, eIF4A and PDCD4 after 1, 2, 4, 6, 24 and 48 hr post-infection by rotavirus and mock infection in Caco2 cells.

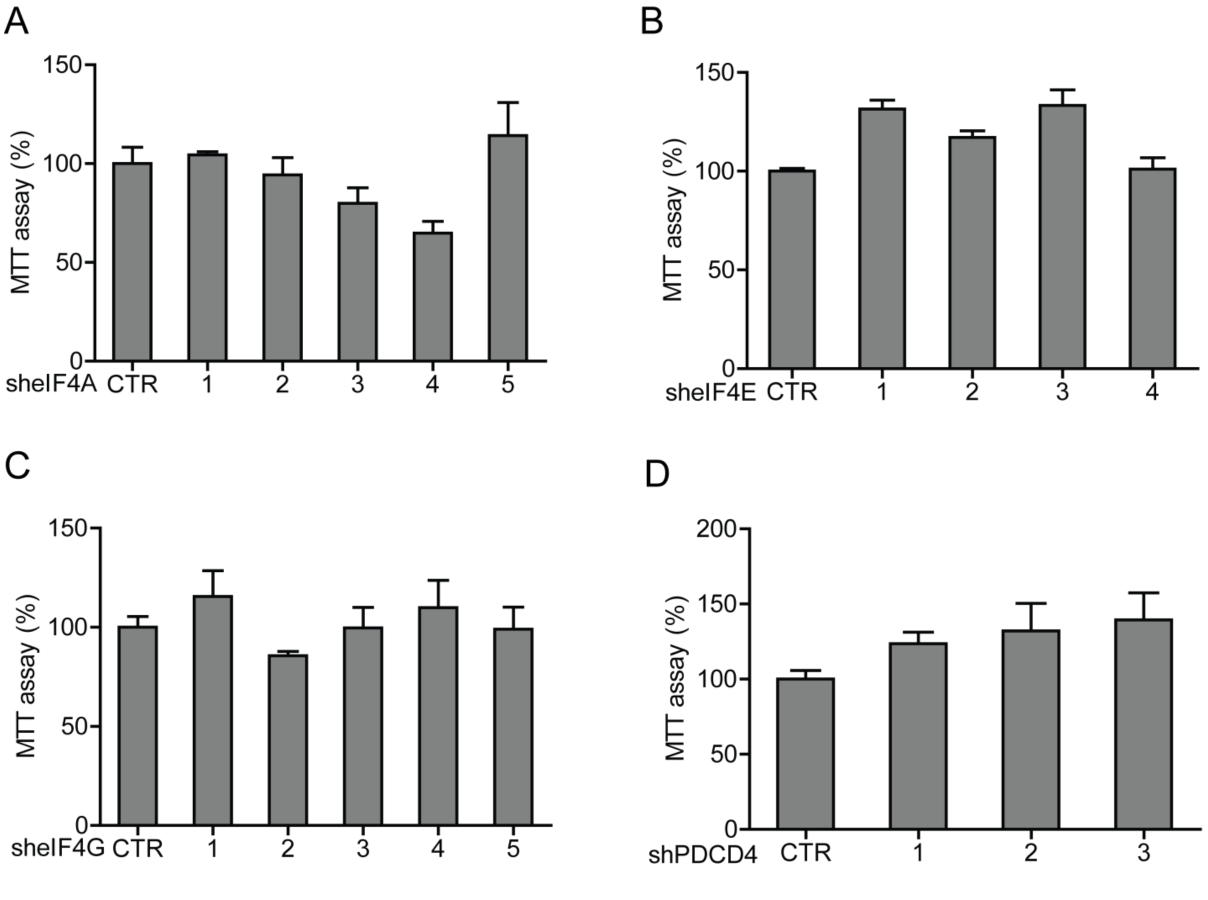

Figure S2. Effect of shRNA against eIF4A (A), eIF4E (B), eIF4G (C) and PDCD4 (D) on host cell viability determined by MTT assays.
